# Review of High Level Endodontic Research in PubMed Index Journals from Iran

**Published:** 2012-08-01

**Authors:** Mohammad Jafar Eghbal, Hassan Torabzadeh

**Affiliations:** 1. Dental Research Center, Shahid Beheshti University of Medical Sciences, Tehran, Iran; 2. Iranian Center for Endodontic Research, Shahid Beheshti University of Medical Sciences, Tehran, Iran

**Keywords:** Biomedical Research, Endodontics, Evidence-Based Dentistry, Iran, Publications, PubMed

## Abstract

**Introduction:**

This study aimed to evaluate patents as well as high level researches including systematic reviews/meta-analyses and randomized controlled clinical trials (RCT) published in scientific journals by Iranians endodontic.

**Materials and Methods:**

The study started with targeted searches of PubMed as well as World Intellectual Property Organization and United State Patent and Trademark Office.

**Results:**

There were 4 filed/granted patents, 2 systematic reviews/meta-analyses and 25 RCTs. Patents were related to endodontic/dental (bio)materials. Performing a topic sorting, 15 RCTs were about vital pulp therapy and 8 about anesthesia and pain. More than 55% of these articles originated from three University of Medical Sciences: Shahid Beheshti (22.2%), Kerman (18.5%) and Mashad (14.8%).

**Conclusion:**

Vital pulp therapy was the most important topic amongst endodontic high level evidence articles.

## Introduction

Evidence-based practice aims to apply the best available evidence gained from scientific methods to clinical decision making. It appraises the strength of evidence of the risks/benefits of treatments as well as diagnostic tests. Evidence-based dentistry (EBD), as a subcategory, is an approach to oral health-care that needs a careful amalgamation of clinically relevant scientific evidence, relating to the patient’s oral condition/history, with the clinician’s skill as well as the patient’s treatment needs/preferences. EBD is about providing personalized dental care based on the current best evidence. The highest level of evidence available represents the current best evidence for a specific clinical question [1] Evidence levels follow a structured hierarchy of criteria for grading the strength of evidence. Some include assessment of a study’s methodological quality, precision of statistical data for the population being studied and other considerations. Although there is no single, universally-accepted hierarchy of evidence, there is broad agreement on the relative strength of the principal types of research. Systematic reviews, meta-analyses and randomized clinical trials rank top while case reports and expert opinions are ranked at the bottom [2][3].

The Oxford Centre for Evidence-based Medicine suggests level A of evidence (LOE1) for Randomized Controlled Clinical Trial (RCT) [4]. RCT is defined as a clinical study in which participants are randomly assigned to either an experimental group or control group. The experimental group receives the new intervention and the control group receives a placebo or standard intervention. These groups are followed for the outcomes of interest [1].

Scientometrics has been employed broadly for assessing the progress of science/technology. It uses articles and patents as important tools to map the development. A recent quantitative scientometric study showed that endodontic publications from different universities in Iran have considerably increased in PubMed index journals [5]. In addition, Iran was in the second rank in the region in year 2010 and the quantitative positive trend in published endodontic articles is considered as a sign of future success in acquiring Iran’s vision by 2025 [6].

However, a science system report from Iran shows some important qualitative weak points such as low expected levels of citations for Life sciences [7]. On the other hand, patent scientometrics is an approach to evaluate the development status of different technology fields. By counting the number of patents (filed or granted), the position of research productivity could be established [8].

In this study, we aimed to evaluate systematic reviews and RCTs as the highest level of evidence which were published in PubMed index journals by Iranian authors as well as patent counting in the field of endodontics.

## Materials and Methods

MESH words searching method without time limitation was utilized for identifying PubMed-indexed endodontic articles [5]. Abstracts were reviewed based on the study design, and unrelated articles were excluded. The data of each article including the first author name/affiliation/publication year/journal name, topic/subject of article, sample size, study period, tested material or technique, and treatment outcomes were extracted.

The World Intellectual Property Organization (WIPO) were searched as the main international and United State Patent and Trademark Office (USPTO) as the most important national agency were searched for Iranian inventors who filed or granted the patents.

## Results

There was only one patent in WIPO, whereas 1 filed and 2 granted patents in USPTO (Table 1). Two systematic reviews about “smear layer” and “root resorption” were found in PubMed index journals (Table 2). The greatest portion of high level evidences (n=25) was related to RCTs (Table 3). Table 4 shows the topics of 25 RCTs. In respect to affiliation grouping, majority of publications belonged to Shahid Beheshti (22.2%),Kerman (18.5%) and Mashad (14.8%) Universities of Medical Sciences (Table 5).

**Table 1:** Four patents in WIPO and USPTO from Iranian endodontists

**Inventor**	**Title**	**Date of application**	**Date of publication**	**Date of approval**	**Sovereign state**	**Number**
Asgary S and Ghassemian Pour Bavandi M	Endodontic filling material	February 22, 2007	28 August 2008	17 June 2012	WIPO	WO/2008/102214
Asgary S	Endodontic filling material	July 16, 2007	August 28, 2008	May 17, 2011	United States	7,942,961 (Patent)
Asgary S	Medical and dental biomaterial and method of use for the same	April 1, 2011	July 28, 2011	January 31, 2012	United States	8,105,086 (Patent)
Saghiri MA *et al.*	Dental cement composition	August 17, 2011	January 19, 2012	-	United States	20120012030

**Table 2:** Systematic reviews by Iranian endodontists

**Author(s)** **Year** **Journal [Ref]**	**Topic**	**Results**
Shahravan *et al.* (2007) JOE [9]	Smear layer	smear layer removal improves the seal
Ahangari *et al.* (2010) CDSR [10]	External root resorption	Lack of high level evidence

**Table 3:** The 25 randomized clinical trials by Iranian endodontists

**Author(s)/Year/Journal/[Ref.]**	**Topic**	**Sample size**	**Time**	**Material(s)/** **Technique**	**Treatment ** **Outcome**
Asgary *et al.*(2012) COI [11]	Pulpotomy (permanent molars)	407	6 and 12 month	CEM Cement *vs.* RCT	CEM pulpotomy>RCT
Asgary and Eghbal (2012) AOS [12]	Pulpotomy (permanent molars)	413	7 day and 12 month	CEM Cement *vs.* MTA	CEM Cement = MTA
Nosrat *et al.(*2012) IJPD [13]	Pulpotomy (immature molars)	118	12 month	CEM Cement *vs.* MTA	CEM Cement = MTA
Eskandarizadeh *et al.*(2011) JCD [14]	Pulp capping (permanent premolars)	90	1, 2 and 3 month	WMTA Vs. GMTA *vs.* Dycal	WMTA = GMTA> Dycal
Zarrabi *et al.*(2011) JOE [15]	Pulp capping (permanent premolars)	32	2 and 8- week	CEM Cement *vs.* MTA	CEM Cement = MTA
Malekafzali *et al.*(2011) EJPD [16]	Pulpotomy (primary molars)	80	6, 12 and 24 month	CEM Cement *vs.* MTA	CEM Cement = MTA
Shahravan *et al.*(2011) IEJ [17]	Pulp capping (third molars)	29	30 day	WMTA (various L/P ratios)	no differences
Zarrabi *et al.*(2010) JOE [18]	Pulp capping (permanent premolars)	32	2 and 8 week	CEM Cement *vs.* MTA	CEM Cement = MTA
Ravanshad *et al.*(2010) JOE [19]	Working length measurement	188	NA	apex locator *vs.* radiograph	no differences
Aminabadi *et al.*(2010) JCPD [20]	Pulp capping (primary molars)	120	2 year	Formocresol *vs.*CH	Formocresol > CH
Parirokh *et al.*(2010) JOE [21]	Anesthesia	150	15 min	Ibuprofen Vs. Indomethacin *vs.* Placebo	Ibuprofen= Indomethacin> Placebo
Asgary and Eghbal (2010) Odont [22]	Pulpotomy/Pain (permanent molars)	407	7-day	CEM Cement *vs.* RCT	CEM pulpotomy>RCT
Ansari and Ranjpour (2010) IEJ [23]	Pulpotomy (primary molars)	40	1-, 6, 12 and 24 month	Formocresol *vs.* MTA	Formocresol = MTA
Jalalzadeh *et al.*(2010) JOE [24]	Postendodontic Pain	40	6, 12, and 24 hours	Prednisolone Vs. placebo	Prednisolone> placebo
Parirokh *et al.*(2010) OOO [25]	Anesthesia	84	During access cavity preparation	Lidocaine Block *vs.* Block+Infiltration	1Block+ 1Infiltration>1Block
Moghadamnia *et al.*(2009) IJDR [26]	Anesthesia	56	0,1, 3, 5, 7, 9 min	Amitriptyline *vs.* Placebo	Amitriptyline > Placebo
Bahrololoomi *et al.*(2008) HJDR [27]	Pulpotomy (primary molars)	70	6 and 9 month	Electrosurgery *vs.*. Formocresol	Electrosurgery = Formocre
Aminabadi *et al.*(2008) JCPD [28]	Pulpotomy (primary incisors)	100	12 and 24 month	Pulpotomy *vs.* RCT	RCT > Pulpotomy
Noorollahian (2008) BDJ [29]	Pulpotomy (primary molars)	60	6, 12 and 24 month	Formocresol *vs.* MTA	Formocresol = MTA
Mehrvarzfar *et al.*(2008) AEJ [30]	Postendodontic Pain	100	6, 12, 24 and 48 h	Dexamethasone *vs.* Placebo	Dexamethasone > Placebo
Aeinehchi *et al.*(2007) IEJ [31]	Pulpotomy (primary molars)	126	3 and 6 month	Formocresol *vs.* MTA	MTA> Formocresol
Ghoddusi *et al.*(2006) NYSDJ [32]	Postendodontic Pain	60	72 hours	1-visit Vs. 2-visit *vs.* 2-visit/CH	
Modaresi *et al.*(2006) OOO [33]	Anesthesia	60	1 hour	Acetaminophen *vs.* Ibuprofen *vs.* Placebo	Ibuprofen> Acetaminophen> Placebo
Mortazavi and Mesbahi (2004) IJPD [34]	RCT (primary teeth)	52	3 and 10 16 month	Vitapex *vs.* ZOE	Vitapex > ZOE
Sadeghein *et al. *(1999) JOE [35]	Pain relief	66	90 min	Ketorolac *vs.*Acetaminophen	Ketorolac>Acetaminophen

**Table 4:** Number of different subjects in RCTs

**Subject**	**Number **
Vital pulp therapy	15
Anesthesia and pain	8
Systematic Review	2
Others	2
**Total**	**27**

**Table 5:** Number of articles from Iranian Universities

**Medical University**	**Number**
Shahid Beheshti	6
Kerman	5
Mashad	4
Azad	2
Shiraz	2
Tabriz	2
Babol	1
Hamedan	1
Rafsanjan	1
Tehran	1
Yazd	1
Zahedan	1
**Total**	**27**

## Discussion

Systematic Review is the gold standard for evidence; it provides a summary of individual studies that have answered the same question; as well as provides a method for managing large quantities of data. It has a clear criteria for retrieval/appraisal/synthesis of evidence from individual RCTs as well as other well-controlled trials. A recent study demonstrated that there were 49 systematic review and meta-analysis in the field of endodontics worldwide [3]. Our results, however, revealed that only 2 systematic reviews (~4%) originated from Iran.

RCTs are regarded as the best study designs to test the efficacy of medical/dental treatments. In RCT the subjects are assigned by chance to separate groups that evaluate new and standard treatments; neither the operators nor the patients can choose which group. At the time of the trial, it is not known which treatment protocol is worse/best. The number of RCTs in the field of dentistry or endodontics has not been reported; however it is estimated that ~4-6% of published articles classified as RCT. Our results showed that RCTs from Iran are ~8% of PubMed-indexed published endodontic articles which seems at or even above the global level.

The aim of our study was to assess the quantity of high-level evidences in endodontics; therefore, we did not appraise the quality of 25 RCTs. It was reported that the quality of RCT in the field of dentistry is still poor, and more efforts for progress are needed. The trials that are not well designed provide biased estimates of the treatment effects; moreover a journal’s impact factor (IF) is not related to the quality of results [35]. Within the outline of EBD, dentists should conscientiously, explicitly, and judiciously use the best current evidence in making decisions about the care of individual patients. Therefore, before trusting RCT reports, a careful evaluationof the reported trial is needed. Randomization as well as blinding, allocation concealment, drop outs analysis are critical quality apparatus of RCTs [35].

Vital pulp therapy (VPT) as well as anesthesia and pain were two main subjects of published Iranian RCTs. VPT in pediatric dentistry is a well established treatment modality. Our data showed that 7 VPT/RCTs were carried out in the field of pediatric to test Formocresol, MTA, Electrosurgery, calcium hydroxide and CEM cement as pulpotomy agents [16][20][23][27][31]. However VPT for mature permanent teeth with sign/symptom of irreversible pulpitis and also carious pulp exposure remains the most challenging areas in endodontics. Our results revealed that 8 VPT/RCTs were carried out for permanent teeth in the field of endodontics to test MTA and CEM cement as pulp capping or pulpotomy biomaterials [11][12][13][14][15][17][18][22]. These RCTs provide a body of evidence that permanent teeth with irreversible pulpitis can be managed successfully by VPT. A recent systematic review confirms this new concept [36].

It was reported that Mashad, Tabriz and Tehran dental schools were the top three institutions when looking at the number of published PubMed-indexed endodontic articles in Iran [4]; however, the present results revealed that Shahid Beheshti, Kerman and Mashad dental schools provide the majority (>55%) of high-level evidences in the field of endodontics.

Relevant literature regarding patents scientometrics in endodontics was found not. However, we found 4 patent documents in the field of endodontics originated from Iran in WIPO and USPTO. There are no patents from other dental subspecialties from Iran.

## Conclusion

It seems that endodontics rank at the first place for science production as well as technology in Iran.
